# Community interventions for people with complex emotional needs that meet the criteria for personality disorder diagnoses: systematic review of economic evaluations and expert commentary

**DOI:** 10.1192/bjo.2021.1043

**Published:** 2021-11-15

**Authors:** Joe Botham, Amy Clark, Thomas Steare, Ruth Stuart, Sian Oram, Brynmor Lloyd-Evans, Tamar Jeynes, Eva Broeckelmann, Mike Crawford, Sonia Johnson, Alan Simpson, Paul McCrone

**Affiliations:** NIHR Mental Health Policy Research Unit, Health Service and Population Research Department, Institute of Psychiatry, Psychology & Neuroscience, King's College London, UK; NIHR Mental Health Policy Research Unit, Health Service and Population Research Department, Institute of Psychiatry, Psychology & Neuroscience, King's College London, UK; NIHR Mental Health Policy Research Unit, Division of Psychiatry, University College London, UK; NIHR Mental Health Policy Research Unit, Health Service and Population Research Department, Institute of Psychiatry, Psychology & Neuroscience, King's College London, UK; NIHR Mental Health Policy Research Unit, Health Service and Population Research Department, Institute of Psychiatry, Psychology & Neuroscience, King's College London, UK; NIHR Mental Health Policy Research Unit, Division of Psychiatry, University College London, UK; NIHR Mental Health Policy Research Unit, Health Service and Population Research Department, Institute of Psychiatry, Psychology & Neuroscience, King's College London, UK; NIHR Mental Health Policy Research Unit, Health Service and Population Research Department, Institute of Psychiatry, Psychology & Neuroscience, King's College London, UK; Division of Psychiatry, Imperial College London, UK; NIHR Mental Health Policy Research Unit, Division of Psychiatry, University College London, UK; NIHR Mental Health Policy Research Unit, Health Service and Population Research Department, Institute of Psychiatry, Psychology & Neuroscience, King's College London, UK; NIHR Mental Health Policy Research Unit, Health Service and Population Research Department, Institute of Psychiatry, Psychology & Neuroscience, King's College London, UK; and Institute for Lifecourse Development, University of Greenwich, London, UK

**Keywords:** Health economics, cost-effectiveness, personality disorder, community care, out-patient treatment

## Abstract

**Background:**

Diagnoses of personality disorder are prevalent among people using community secondary mental health services. Identifying cost-effective community-based interventions is important when working with finite resources.

**Aims:**

To assess the cost-effectiveness of primary or secondary care community-based interventions for people with complex emotional needs who meet criteria for a diagnosis of personality disorder to inform healthcare policy-making.

**Method:**

Systematic review (PROSPERO: CRD42020134068) of databases. We included economic evaluations of interventions for adults with complex emotional needs associated with a diagnosis of personality disorder in community mental health settings published before 18 September 2019. Study quality was assessed using the CHEERS statement.

**Results:**

Eighteen studies were included. The studies mainly evaluated psychotherapeutic interventions. Studies were also identified that evaluated altering the setting in which care was delivered and joint crisis plans. No strong economic evidence to support a single intervention or model of community-based care was identified.

**Conclusions:**

Robust economic evidence to support a single intervention or model of community-based care for people with complex emotional needs is lacking. The strongest evidence was for dialectical behaviour therapy, with all three identified studies indicating that it is likely to be cost-effective in community settings compared with treatment as usual. More robust evidence is required on the cost-effectiveness of community-based interventions on which decision makers can confidently base guidelines or allocate resources. The evidence should be based on consistent measures of costs and outcomes with sufficient sample sizes to demonstrate impacts on these.

Globally, it is estimated that approximately 8% of the population experience complex emotional needs that meet the diagnostic criteria for personality disorder, which is described broadly as an enduring and pervasive pattern of emotional and cognitive difficulties that affect the way in which a person relates to others or understands themselves.^1^ The diagnosis is often associated with high rates of psychiatric comorbidity,^2^ high levels of service use^3^ and high treatment costs.^4^

In European and North American community secondary mental healthcare settings, the prevalence of personality disorder diagnoses is estimated to be above 40%.^5^ A range of psychological therapies show some evidence of effectiveness; dialectical behaviour therapy (DBT) and mentalisation-based treatment (MBT) have the most well-established evidence base.^6,7^ Although establishing clinical effectiveness of psychological therapies and models of care is crucial, it is also important for decision makers to consider their value for money. Health and social care resources are limited and there are competing demands for scarce resources. It is therefore a growing requirement that assessments of new treatments and therapies include an economic evaluation.^8^ Through robust economic evaluation decision makers can consider the opportunity cost of funding one intervention over another, as for every potential gain from a funded intervention, given a limited funding pool, there are potential losses from the next best alternative option that is forgone.^9^

Economic evaluation seeks to compare the costs of an intervention against its outcomes. The main approaches are cost-effectiveness analysis (CEA), cost–benefit analysis (CBA), cost–consequences analysis (CCA) and cost–utility analysis (CUA).^10^ CEA compares the incremental cost of an intervention with incremental changes in outcomes usually using a condition-specific measure. For complex emotional needs this may include an assessment of functioning or distress. CBA measures outcomes using monetary units and is uncommon in healthcare evaluations, partly owing to the challenge of valuing health effects in monetary terms. CCA presents costs against a number of outcome measures to support multi-criterion decision-making. Finally, CUA is a type of CEA and it compares the incremental cost of an intervention with changes in a measure of health that allows comparisons across illness areas. The measure used is usually the quality-adjusted life-year (QALY), where one QALY is equivalent to 1 year of life in perfect health. Results can be compared against a willingness to pay (WTP) threshold which indicates how much a society will pay for a QALY. The National Institute for Health and Care Excellence (NICE) uses a threshold of up to £30 000 in England.^11^

We are aware of two systematic reviews of economic evaluations of interventions for personality disorder.^3^ The scope of these reviews was limited to interventions for people with a diagnosis of borderline personality disorder only and the most recent papers included were published in 2014. The previous reviews included cost comparisons and interventions delivered in non-community settings. The aim of this paper is to assess the cost-effectiveness of community-based interventions for people with complex emotional needs that meet criteria for diagnoses of personality disorder compared with usual care (as defined by each study) or other active interventions and to include more recent evaluations. To do this we systematically review and assess the quality of relevant economic evaluations. The review forms part of a wider programme of work on complex emotional needs funded by the National Institute for Health Research in England. As is standard practice with our programme of work, we have included two independent commentaries on the review from people with lived experience.

## Method

This systematic literature review was carried out in accordance with the Preferred Reporting Items for Systematic Reviews and Meta-Analyses (PRISMA) checklist.^12^ A protocol for the search strategy and methods has been registered with PROSPERO (CRD42020134068). We use ‘complex emotional needs’ as our preferred terminology rather than ‘personality disorder’. This choice seeks to recognise that, although some people find the diagnostic term helpful, many find it to be invalidating and stigmatising. However, we do use the term personality disorder when referring to original papers and search strategy inclusion criteria (diagnostic inclusion criterion for the service model described), as appropriate.

### Eligibility criteria

Inclusion criteria (detailed in the Appendix near the end of this paper) required that studies: (a) included an economic evaluation where both costs and outcomes are reported and where a formal or informal link between costs and outcomes is made; (b) included adults attending a general or specialist community mental health service with complex emotional needs that meet the diagnostic criteria for personality disorder (other than antisocial personality disorder); (c) evaluated community-based services, treatments and interventions; and (d) compared cost-effectiveness with usual care or another active intervention.

Publication date was limited to the period from 1 January 1990 to 18 September 2019, and language of publication to English. Studies of interventions for adults with diagnosed antisocial personality disorder were excluded, as there are often high levels of forensic service use, which would limit or prevent the implementation of community-based interventions. Cost-minimisation studies were excluded as they require interventions to have an equivalent effect, which would be incredibly unlikely in this setting. Community-based services, treatments and interventions are defined as any services, treatments and interventions provided outside of an in-patient setting and excluding pharmacological treatments.

### Information sources and search terms

Electronic searches of the following bibliographic databases were conducted: MEDLINE, PsycInfo, Embase, Global Health and the NHS Economic Evaluation Database (NHS EED). Search terms can be found in the supplementary Appendix, available at https://doi.org/10.1192/bjo.2021.1043. Search domains covered are: ‘personality disorder’, ‘community care’ and ‘economic evaluation’.

### Selection process and data collection

The search was first run on 18 September 2019. Three authors (J.B., T.S. and R.S.) independently screened titles and abstracts against the inclusion criteria using the systematic review web application Rayyan QCRI (Rayyan Systems Inc., State of Qatar; see www.rayyan.ai), which has a masking (‘blinding’) feature. A fourth author (P.M.) independently reviewed and resolved disagreements. Full-text screening was conducted by J.B. and T.S. independently (with P.M. resolving disagreements) using eligibility forms pertaining to each aspect of the inclusion criteria. Reviewers were not masked to authors, institutions or journals. We did not conduct a formal test for interrater reliability.

Data were extracted into standard forms by J.B.. Duplicate extraction was completed by T.S. for 25% of papers and P.M. resolved disagreements. Data were collected on the characteristics, methods and results of each study. The study characteristics data included the country, funder, intervention (including levels of contact), the comparator, study design, economic approach (i.e. whether a CEA or CUA was used), a summary of the inclusion and exclusion criteria, the sample size and sample characteristics (mean age, proportion female and proportion employed).

Data on the study methods included the follow-up period, perspective, costing (approach to recording service use, valuing the intervention, sources of unit costs and discounting) and outcomes (main economic outcome, QALY derivation if relevant and discounting). The perspective is the range of costs included in an analysis. A narrow perspective would only include costs relevant to the service provider. A societal perspective would include costs to other parts of society, such as the criminal justice system, social services, a patient's employer or voluntary/informal care. Inclusion of all impacts would be a perspective that is rarely achieved. In this paper, we generally use the term ‘broad’ to define a perspective that is beyond the health and social care sector. Resource use measurement methods include reviewing hospital records or conducting patient interviews/surveys. The source of unit costs includes national routine data, information from local providers, and fees or tariffs. The outcome is the measure of effectiveness used in the evaluation. This can be specific to a condition, such as an improvement on a condition-specific scale, or generic, such as QALYs. For both costs and outcomes, data on any discounting has been recorded. Discounting is used to reflect the perceived lower value attached to future cost and outcomes, owing to the widely accepted view of positive time preference. It is conventional to discount only costs and outcomes that occur beyond a 1-year period.^9^

### Data analysis

Meta-analysis was not undertaken as the economic evaluations are context specific and the review does not focus on one particular outcome. Narrative synthesis was undertaken to describe the main study findings based on the reporting of the data items described above, including outcomes and costs for the intervention and comparator groups, and the cost–utility or cost-effectiveness results. Results were often expressed as incremental cost-effectiveness or cost–utility ratios. These ratios are typically derived from mean values and, owing to variation around the mean, consideration should be given to uncertainty in these estimates.

### Quality assessment

Study quality was assessed using the Consolidated Health Economic Evaluation Reporting Standards (CHEERS) statement, which sets out a standard for the reporting of economic evaluations.^13^ The statement evaluates the quality of reporting rather than the quality of evidence. Two statements relate to the introduction, sixteen to the methods, five to the results, and there is one statement each for the title, abstract, sources of funding and conflicts of interest. J.B. appraised 50% of papers and A.C. appraised the remaining 50%. T.S. provided an independent appraisal of 25% of papers and any disagreements were resolved by P.M. Each paper is assigned a score, which corresponds to how many of the 28 statements on the CHEERS checklist the paper complies with (0, does not comply; 0.5, partially complies; 1, fully complies). Some of the statements in the checklist relate to the quality and fullness of reporting, which can be influenced by publications (e.g. the structure of the abstract) rather than the quality of the research itself. A breakdown of each paper's score is reported, so scores for methodology and results can be assessed independently.

## Results

**Fig. 1:**
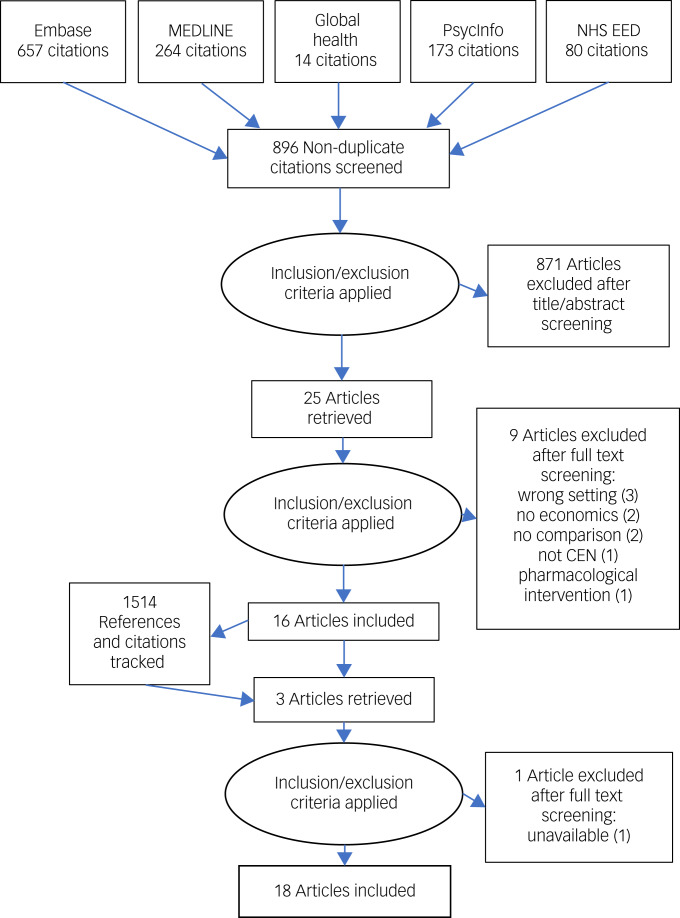
PRISMA flowchart. CEN, complex emotional needs.

Figure 1 shows the PRISMA flowchart pertaining to the review; 18 papers were included in the review. One paper presented both CEAs and CUAs for each of two separate interventions.^14^ We report on these interventions separately although both were compared with the same control group.

P.M. resolved 14 disagreements between J.B. and T.S. during title and abstract screening; 2 during full-text screening; 14 over data extraction; and disagreements over 4 papers in the quality assessment.

### Study characteristics

Table 1 and Table 2 show the characteristics of the studies included in the review.

**Table 1:** Study characteristics

Study and country	Intervention	Quantity	Comparison	Design	Economic evaluation method	*n*	Mean age, years	Female, %	Employed, %
Murphy et al,^15^ Ireland	DBT	12 months, weekly sessions	TAU	BAA	CUA	196	−	81.0	21.0
Pasieczny & Connor,^16^ Australia	DBT	6 months, weekly individual sessions of 1 h, weekly group sessions of 2 h, out-of-hours phone access	TAU	RCT	CCA	DBT: 43 TAU: 47	DBT: 33.6 TAU: 33.2	DBT: 95.0 TAU: 92.0	DBT: 21.0 TAU: 21.0
Priebe et al,^17^ UK	DBT	12 months, weekly individual sessions of 1 h, weekly group sessions of 2 h, out-of-hours phone access	TAU	RCT	CEA	DBT: 40 TAU: 40	DBT: 33.0 TAU: 31.3	DBT: 87.5 TAU: 87.5	DBT: 35.0 TAU: 37.5
Davidson et al,^18^ UK	CBT	12 months, 30 sessions of 1 h	TAU	RCT	CCA	CBT: 43 TAU: 33	CBT: 32.4 TAU: 31.4	CBT: 83.3 TAU: 84.6	CBT: 68.5 TAU: 67.3
Palmer et al,^19^ UK	CBT	27 sessions offered	TAU	RCT	CUA	CBT: 54 TAU: 52	CBT: 32.4 TAU: 31.4	CBT: 83.3 TAU: 84.6	CBT: 68.5 TAU: 67.3
Tyrer et al,^20^ UK	MACT	Booklet and 7 sessions	TAU	RCT	CCA	MACT 239 TAU: 241	31.0	68.0	−
Grenyer et al,^21^ Australia	SC	18 months, 3 sessions/month of 30 min	TAU	RCT	CCA	SC: 335 TAU: 307	SC: 36.6 TAU: 37.1	SC: 46.0 TAU: 55.4	−
Kvarstein et al,^22^ Norway	SC	3 phases of varying intensity delivered over 4 years	OP	RCT	CEA	SC: 56 OP: 51	31.0	76.0	−
Sinnaeve et al,^23^ Netherlands	SC	3 months of residential DBT, 6 months of out-patient DBT	DBT-OP	RCT	CUA	DBT-I: 39 DBT-OP: 16	DBT-I: 26.2 DBT-OP: 25.6	95.0	26.0
Ranger et al,^24^ UK	NID	Up to 15 sessions	AO	RCT	CEA	NID: 26 AO: 22	−	NID: 39.0 AO: 25.0	−
Tyrer et al,^25^ UK	NID	Up to 15 sessions	AOR	RCT	CCA	NID: 19 AOR: 15	−	NID: 39.0 AOR: 25.0	−
Bamelis et al,^14^ Netherlands	ST	40 sessions/week in year 1, 10 booster sessions in year 2	TAU	RCT	CEA CUA	ST: 145 TAU: 134	ST: 37.6 TAU: 38.1	ST: 54.5 TAU: 59.0	ST: 45.5 TAU: 47.0
Van Asselt et al,^26^ Netherlands	ST, TFP	ST: 36 months, 2 sessions/week of 50 min TFP 36 months, 2 sessions/week of 50 min	−	RCT	CUA	ST: 44 TFP: 42	ST: 31.7 TFP: 29.5	ST: 90.9 TFP: 95.2	ST: 20.5 TFP: 19.0
Soeteman et al,^27^ Netherlands	OP, DH, IP	OP: 15 months, up to 2 weekly sessions DH: 10 months, 1–5 sessions/week IP: 9 months, 5–7 sessions/week	−	Markov Model (of trial data)	CUA	OP: 57 DH: 99 IP: 85	OP: 35.4 DH: 31.4 IP: 28.9	OP: 64.9 DH: 76.8 IP: 70.6	−
Soeteman et al,^28^ Netherlands	LOP, SDH, LDH, SIP, LIP	LOP: ≤6 months, 2 sessions per week SDH: ≤6 months, 1–5 sessions per week LDH: >6 months, 1–5 sessions per week SIP: ≤6 months, 5–7 sessions per week LIP: >6 months, 5–7 sessions per week	−	Markov Model (of trial data)	CUA	LOP: 96 SDH: 85 LDH: 103 SIP: 63 LIP: 101	LOP: 36.2 SDH: 35.0 LDH: 31.9 SIP: 37.6 LIP: 28.4	LOP: 66.7 SDH: 77.6 LDH: 75.7 SIP: 61.9 LIP: 65.3	−
Borschmann et al,^29^ UK	JCP	2 planning sessions of 1 h each	TAU	RCT	CUA	JCP: 46 TAU: 42	JCP: 35.6 TAU: 36.1	JCP: 78.3 TAU: 83.3	JCP: 13.0 TAU: 9.5
McMurran et al,^30^ UK	PEPS	12 group sessions of 2 h	TAU	RCT	CUA	PEPS: 154 TAU: 152	PEPS: 38.6 TAU: 37.8	PEPS: 75.0 TAU: 76.0	PEPS: 32.0 TAU: 37.0
Bamelis et al,^14^ Netherlands	COP	Open ended weekly sessions	TAU	RCT	CEA CUA	COP: 41 TAU: 75	COP: 39.2 TAU: 38.1	COP: 56.1 TAU: 59.0	COP: 39.0 TAU: 47.0
Blankers et al,^31^ Netherlands	MBT	18 months intensive day hospital programme (6 h/day, 5 days/week followed by 18 months of group therapy	TAU	RCT	CEA CUA	MBT: 54 TAU: 41	MBT: 34.3 TAU: 34.0	MBT: 77.0 TAU: 54.5	MBT: 26.0 TAU: 12.5

ST, schema therapy; COP, clarification-oriented psychotherapy; MBT, mentalisation-based therapy; JCP, joint crisis plans; CBT, cognitive–behavioural therapy; SC, stepped care; PEPS, psychoeducation with problem-solving; DBT, dialectical behaviour therapy; NID, nidotherapy; DBT-I, in-patient DBT; DBT-OP, out-patient DBT; DH, day hospital; LOP, long-duration OP; SDH, short-duration DH; LDH, long-duration DH; SIP, short-duration IP; LIP, long-duration IP; MACT, manual-assisted cognitive therapy; TFP, transference-focused psychotherapy; TAU, treatment as usual; AO, assertive outreach; AOR, assertive outreach and rehabilitation; CEA, cost-effectiveness analysis; CUA, cost-utility analysis.

**Table 2:** Further study characteristics

Study and country	Follow-up period	Costing perspective	Service use measure	Unit costs	Unit costs	Cost discount rate	Main economic outcome	QALY measure	QALY discount rate
Murphy et al,^15^ Ireland	18	Health service	Patient-reported ‘dedicated resource use questionnaire’	Micro-costing approach	DRG estimates, literature and national averages	−	QALYs	EQ-5D (Irish tariff)	−
Pasieczny & Connor,^16^ Australia	6	Health service	Hospital records	Micro-costing approach	Local costs	−	NSSI, ED visits, frequency and length of in-patient stays, BDI-II, BSS, STAI-Y and BSI	n.a.	−
Priebe et al,^17^ UK	12	Broad	Patient reported CSRI		National averages	−	Percentage point difference in self-harm rates	n.a.	−
Sinnaeve et al,^23^ Netherlands	12	Broad	Patient-reported TiC-P	−	National averages	−	QALYs	EQ-5D (Dutch tariff)	−
Davidson et al,^18^ UK	72	Health, social care & criminal justice	Combination of patient-reported CSRI and hospital records	−	−	−	QALYs	EQ-5D (UK tariff)	−
Palmer et al,^19^ UK	24	Broad	Combination of patient-reported CSRI and hospital records	Micro-costing approach	Local/national data-sets	3.5%	QALYs - discounted at 3.5% annually.	EQ-5D (UK tariff)	4%
Tyrer et al,^20^ UK	12	Broad	Patient-reported CSRI	−	National averages	−	Linehan's Parasuicide History Interview	n.a.	−
Grenyer et al,^21^ Australia	36	Inpatient costs	Patient/hospital records	−	National average.	−	Frequency & length of in-patient stays.	n.a.	−
Kvarstein et al,^22^ Norway	48	Health & social care	Patient-reported	Top-down.	National averages.	−	GAF	n.a.	−
Ranger et al,^24^ UK	12	Broad	Patient-reported SFSUS	Micro-costing approach	National averages.	−	BPRS	n.a.	−
Tyrer et al,^25^ UK	12	Broad	Patient-reported SFSUS	Micro-costing approach.	National averages.	−	Bed-days	n.a.	−
Bamelis et al,^14^ Netherlands	36	Broad	Combination of patient-reported and hospital records	-	National averages	4%	QALYs and SCID-II	EQ-5D (UK tariff)	−
Van Asselt et al,^26^ Netherlands	48	Broad	Patient-reported interview	Micro-costing approach.	National averages.	4%	QALYs	EQ-5D (UK tariff)	4%
Soeteman et al, a^27^ Netherlands	60	Broad	Combination of patient-reported TiC-P and hospital records	Micro-costing approach.	National averages.	−	QALYs and recovered patient years	EQ-5D (Dutch tariff)	−
Soeteman et al, b^28^ Netherlands	60	Broad	Combination of Patient-reported TiC-P and hospital records	Micro-costing approach.	National averages.	3%	QALYs and recovered patient years	EQ-5D (Dutch tariff)	−
Borschmann et al,^29^ UK	6	Health and social Care	Combination of patient-reported AD-SUS and hospital records	Micro-costing approach	National averages.	−	QALYs	EQ-5D (UK tariff)	−
McMurran et al,^30^ UK	72	Broad	Patient-reported CSRI	Micro-costing approach	National averages	−	QALYs	EQ-5D (Irish tariff)	−
Bamelis et al,^14^ Netherlands	36	Broad	Combination of patient-reported and hospital records	−	National averages	4%	QALYs and SCID-II	EQ-5D (UK tariff)	−
Blankers et al,^31^ Netherlands	36	Broad	TiC-P	−	National averages	4%	QALYs (discounted at 1.5%) and BPDSI <15	EQ-5D (Dutch tariff)	1.5%

DRG, diagnosis-related group; QALY, quality adjusted life year; EQ-5D, EuroQol's EQ-5D; n.a., not applicable; NSSI, non-suicidal self-injury; ED, emergency department; BDI-II, Beck Depression Inventory II; BSS, Beck Scale for Suicidal Ideation; STAI-Y, Spielberger State–Trait Anxiety Inventory Form Y; BSI, Brief Symptom Inventory; CSRI, Client Service Receipt Inventory; TiC-P, Treatment Inventory Cost in Psychiatric Patients; GAF, Global Assessment of Functioning; BPRS, Brief Psychiatric Rating Scale; SFSUS, Secure Facilities Use Schedule; SCID-II, Structured Clinical Interview for DSM-IV for Axis II Disorders; AD-SUS, Adult Service Use Schedule; BPDSI, Borderline Personality Disorder Severity Index.

Studies were conducted in the UK, Europe and Australia, with sample sizes ranging from 34 to 642 (mean 195.2, s.d. = 167.7). The youngest sample had a mean age of 28.4 years and the oldest sample's mean was 39.2 years. The percentage of women in the samples ranged from 25 to 95%. The lowest rate of employment in a sample was 9.5% and the highest rate of employment was 68.5%, though seven studies did not report this.

Five of the studies included patients with any personality disorder diagnosis.^17,21,22,25,30^ Three of the evaluations looked at cluster C personality disorders, described as avoidant, dependent or obsessive–compulsive,^14,28^ and one study looked at cluster B personality disorders, described as antisocial, borderline, histrionic or narcissistic.^27^ Seven studies only recruited participants with a diagnosis of borderline personality disorder.^16,^^18,19,23,26,29,31^ Nine of the studies required a recent episode of self-harm, attendance at an emergency department or in-patient stay.^15,17–21,23,29^ Two studies only included patients with other comorbid severe mental illness^24^ and one of the two also required substance dependence.^25^ One study included a general sample of which less than half had complex emotional needs that met personality disorder diagnostic criteria.^20^

The interventions evaluated in the 18 studies are described in the supplementary Table 1. Psychotherapeutic interventions included dialectical behaviour therapy (*n* = 3), types of cognitive therapy (*n* = 3), nidotherapy (*n* = 2), schema-focused therapy (*n* = 2), psychoeducation with problem-solving (*n* = 1) and mentalisation-based therapy delivered in a day-hospital setting (MBT) (*n* = 1). Other interventions involved altering the setting in which care was delivered (*n* = 2), adopting a stepped-care approach (*n* = 3) and developing joint crisis plans (*n* = 1). Treatment as usual was the comparative intervention in eight of the included papers. Other comparators included psychotherapy provided by out-patient services (*n* = 4), psychotherapy provided by day-hospital services (*n* = 2), care provided by assertive outreach services (*n* = 2), transference-focused psychotherapy (*n* = 1), and baseline data (*n* = 1). More than three-quarters of the included papers employed a randomised controlled trial (RCT) design (*n* = 14), two used Markov models, one a wait-list controlled trial and one a quasi-experimental design.

In total, 33 different outcome measures were used across the 18 studies (Table 3), with inconsistent approaches to discounting outcomes (5 studies applied a discount rate of between 3 and 5% and 13 studies applied no discounting). Over half of the papers employed a CUA (*n* = 10). Other approaches used were CCAs (*n* = 5) and CEAs (*n* = 5).

**Table 3:** Outcomes used in studies

Outcome	Measure(s)	Papers	Prim/Sec
Recovered patients	SCID-II	Bamelis et al^14^	Primary
		Davidson et al^18^	
	ADP-IV	Bamelis et al^14^	Primary (back-up)^a^
	BPDSI	Blankers et al^31^	Effectiveness measure
		van Asselt et al^26^	
HRQoL	EQ-5D-3L	Bamelis et al^14^	Economic outcome
		Blankers et al^31^	Utility measure
		Borschmann et al^29^	Secondary
		McMurran et al^30^	
		Palmer et al^19^	Primary
		Davidson et al^18^	
		Sinnaeve et al^23^	
		Soeteman et al^27^	
		Soeteman et al^28^	
		van Asselt et al^26^	
	EQ-5D-5L	Murphy et al^15^	
Self-harm/non-suicidal self-injury	Self-report questionnaire	Borschmann et al^29^	Primary
	ADSHI	Davidson et al^18^	
	Structured interview	Priebe et al^17^	Primary
		Pasieczny & Connor^16^	Clinical service measure
	LPC	Sinnaeve et al^23^	
	LPHI	Tyrer et al^20^	
Depression and anxiety	HADS	Borschmann et al^29^	Secondary
Working alliance	WAI	Borschmann et al^29^	Secondary
Client satisfaction	CSQ	Borschmann et al^29^	Secondary
Client engagement	SES	Borschmann et al^29^	Secondary
	EAS	Tyrer et al^25^	
Mental well-being	WEMWS	Borschmann et al^29^	Secondary
Social functioning	WSAS	Borschmann et al^29^	Secondary
	SFQ	Davidson et al^18^	
		Ranger et al^24^	Secondary
		Tyrer et al^25^	Secondary
	IIP-32	Davidson et al^18^	
Coercion (during hospital visits)	TES	Borschmann et al^29^	Secondary
Frequency of in-patient visits	−	Grenyer et al^21^	Primary
		Pasieczny & Connor^16^	Clinical service measure
Duration of in-patient visits	−	Grenyer et al^21^	Primary
		Pasieczny & Connor^16^	Clinical service measure
		Ranger et al^24^	Primary
		Tyrer et al^25^	Primary
Frequency of emergency department visits	−	Grenyer et al^21^	Primary
		Pasieczny & Connor^16^	Clinical service measure
Functioning	GAF	Kvarsein et al^22^	
Depression	BDI	Davidson et al^18^	
		Pasieczny & Connor^16^	Self-report measure
Anxiety	STAI	Davidson et al^18^	
		Pasieczny & Connor^16^	Self-report measure
Beliefs related to personality disorder	YSQ	Davidson et al^18^	
Suicide attempts	−	Pasieczny & Connor^16^	Clinical service measure
	LPC	Sinnaeve et al^23^	
Behavioural and service use	−	Pasieczny & Connor^16^	Clinical service measure
Suicidal planning	BSSI	Pasieczny & Connor^16^	Self-report measure
Psychiatric symptoms	BSI	Pasieczny & Connor^16^	Self-report measure
		Priebe et al^17^	Secondary
	BPRS	Priebe et al^17^	Secondary
		Ranger et al^24^	Secondary
		Tyrer et al^25^	Secondary
Borderline personality disorder symptoms/severity	ZRS-BPD	Priebe et al^17^	Secondary
	BPDSI	Sinnaeve et al^23^	
Subjective QoL	MSA-QoL	Priebe et al^17^	Secondary
Recovered patient years	−	Soeteman et al^27^	
		Soeteman et al^28^	
Personality status	PAS-Q	Tyrer et al^20^	

a. If data for the primary outcome were missing, back-up data were used instead.

SCID-II, Structured Clinical Interview for DSM-IV Axis II Disorders; ADP-IV, Assessment of DSM-IV Personality Disorder Questionnaire; BPDSI, Borderline Personality Disorder Severity Index; EQ-5D-3L, EuroQuol EQ-5D-3L; EQ-5D-5L, EuroQuol EQ-5D-5L; ADSHI, Acts of Deliberate Self Harm; LPC, Life-time Parasuicide Count; LPHI, Lineham's Parasuicide History Interview; HADS, Hospital Anxiety and Depression Scale; WAI, Working Alliance Inventory; CSQ, Client Satisfaction Questionnaire; SES, Service Engagement Scale; EAS, Engagement and Assessment Scale; WEMWS, Warwick–Edinburgh Mental Well-being Scale; WSAS, Work and Social Adjustment Scale; SFQ, Social Functioning Questionnaire; IIP-32, Inventory of Interpersonal Problems - Short Form 32; TES, Treatment Experience Scale; GAF, Global Assessment of Functioning; BDI, Beck Depression Inventory; STAI, Spielberger State–Trait Anxiety Inventory; YSQ, Young Schema Questionnaire; BSSI, Beck Scale for Suicidal Ideation; BSI, Brief Symptom Inventory; BPRS, Brief Psychiatric Rating Scale; ZRS-BPD, Zanari Rating Scale for Borderline Personality Disorder; MSA-QoL, Manchester Short Assessment of Quality of Life; PAS-Q, Quick Personality Assessment Schedule.

Outcomes and costs reported by all studies are reported in Table 4.

**Table 4:** Study results

Study and country	Intervention group cost	Comparison group cost	Intervention group outcome	Comparison group outcome	Main economic result
Murphy et al,^15^ Ireland	€16 514	€16 266	0.69 QALYs	0.49 QALYs	€1965 per QALY for DBT compared with TAU
Pasieczny & Connor^16^ Australia	Au$12 196	Au$18 123	2.23 bed-days	13.60 bed-days	DBT less costly and leads to fewer bed-days compared with TAU
Priebe et al,^17^ UK	£5685 (£6792 including lost work)	£3754 (£4786 including lost work)			£36 per 1% point reduction in incidence of self-harm for DBT compared with TAU
Sinnaeve et al,^23^ Netherlands	€19 899	€12 427	0.65 QALYs	0.62 QALYs	€278 067 per QALY with stepped-care DBT compared with out-patient DBT
Davidson et al,^18^ UK	£6582	£18 737	0.46 QALYs	0.61 QALYs	CBT-PD estimated to be cost saving but achieve fewer QALYs than TAU
Palmer et al,^19^ UK	£12 785	£18 356	1.06 QALYs	1.20 QALYs	CBT less costly but also less effective than TAU
Tyrer et al,^20^ UK	£14 524	£15 665	Incidence rate of parasuicide: 0.584 per year	Incidence rate of parasuicide: 0.71 per year	MACT has similar costs and lower proportion experiencing outcome compared with TAU
Grenyer et al,^21^ Australia	$3774	$7435	4.28 mean bed-days	8.44 mean bed-days	Stepped-care estimated to be cheaper than TAU, with fewer bed-days
Kvarstein et al,^22^ Norway	€31,823	€31,607	10-point improvement in GAF score	18-point improvement in GAF score	€1092 per one point GAF improvement for avoidant personality disorder receiving out-patient care compared with stepped care
Ranger et al,^24^ UK	£23 796	£27 908	24.8 BPRS score	29.2 BPRS score	Nidotherapy dominant compared with assertive outreach
Tyrer et al,^25^ UK	£18 963	£33 668	54 bed-days	139 bed-days	Fewer bed-days and lower costs with nidotherapy than with assertive outreach and rehabilitation service
Bamelis et al,^14^ Netherlands	€23 805	€26 333	2.34 QALYs	2.23 QALYs	Schema therapy dominant compared with TAU
Van Asselt et al,^26^ Netherlands	€37 826	€46 795	2.15 QALYs 52% recovered	2.27 QALYs 29% recovered	<90% probability ST is cost-effective for QALYs and recovered patients. As WTP per QALY increases, % cost-effectiveness decreases (at a threshold of €20 000, ST has 84% probability of being cost-effective). As WTP per recovered patient increases, probability of cost-effectiveness does too
Soeteman et al, a^27^ Netherlands	In-patient €97 351 Day hospital €91 090	Out-patient €80 247	In-patient 3.32 Day hospital 3.30	Out-patient 3.11	Out-patient care most likely to be cost-effective compared with in-patient and day hospital for cluster B
Soeteman et al, b^28^ Netherlands	Short term in-patient €91 620 Long-term in-patient €119 946 Short-term day hospital €89 411 Long-term day hospital €105 940	Long-term outpatient €89 936	Short-term in-patient: 3.57 QALYS Long-term in-patient: 3.49 QALYs Short-term day hospital: 3.44 QALYs Long-term day hospital: 3.49 QALYs	Long-term out-patient: 3.30 QALYs	All long-term treatments dominated short-term in-patient €16 570/QALY compared with short-term day hospital for cluster C
Borschmann et al,^29^ UK	£5308	£5631	0.31 QALYs	0.30 QALYs	Joint crisis plans dominant compared with. TAU
McMurran et al,^30^ UK	£6777	£8072	0.56 QALYs	0.57 QALYs	Psychoeducation with problem-solving has a 64% chance of being more cost-effective at a threshold of £20 000–£30 000 per QALY compared with TAU
Bamelis et al,^14^ Netherlands	€30 070	€26 333	2.23 QALYs	2.23 QALYs	Clarification-orientated psychotherapy dominated by TAU
Blankers et al,^31^ Netherlands	€64 121	€61 141	1.3 QALYs	1.5 QALYs	MBT dominated by TAU

QALY, quality-adjusted life year; DBT, dialectical behaviour therapy; TAU, treatment as usual; CBT-PD, cognitive–behavioural therapy for personality disorders; CBT, cognitive–behavioural therapy; MACT, manual-assisted cognitive therapy; GAF, Global Assessment of Functioning; BPRS, Brief Psychiatric Rating Scale; ST, schema therapy; WTP, willingness to pay; MBT, mentalisation-based treatment.

### Dialectical behaviour therapy

Each of the three studies of DBT used a different method of economic evaluation. Murphy et al conducted a quasi-experimental study using a CUA,^15^ Pasieczny & Connor adopted a CCA for their waiting-list controlled trial^16^ and Priebe et al performed a CEA based on an RCT.^17^ The mean follow-up period for these studies was 12 months (s.d. = 6 months). Murphy et al and Pasieczny & Connor adopted a narrow healthcare perspective, whereas Priebe et al took a broader view including lost employment costs.

Two of the studies^16,17^ reported significant improvements in clinical outcomes. Pasieczny & Connor reported fewer suicide attempts, emergency department visits, admissions and in-patient days.^16^ Priebe et al reported a reduction in self-harm events (rate ratio RR = 0.78; 95% CI 0.76–0.80).^17^ Pasieczny & Connor and Murphy et al reported DBT to be less costly than the control condition, although neither reported statistical significance.^15^ Murphy et al reported an incremental cost-effectiveness ratio (ICER) for DBT of €1965 per QALY, with the sensitivity analysis suggesting a 62% probability that DBT was cost-effective at a threshold (i.e. how much society is considered to value a QALY) of €45 000 EUR per QALY. Priebe et al reported the ICER for DBT to be £36 per percentage point decrease in the incidence of self-harm.^17^

### Cognitive therapies

All three studies evaluating cognitive therapies used data from RCTs. Palmer et al carried out a CUA, whereas Davidson et al and Tyrer et al employed a CCA.^18–20^ Tyrer et al evaluated manual-assisted cognitive therapy (MACT), with the other two evaluating cognitive–behavioural therapy (CBT). The follow-up periods for these studies were the most varied for a specific intervention category (mean 36 months, s.d. = 31.75). Tyrer et al and Palmer et al took a broad perspective and Davidson et al employed a health, social care and criminal justice perspective. All three used a version of the Client Service Receipt Inventory to record resource use, although Davidson et al and Palmer et al also used hospital records.

Only Tyrer et al reported a change in outcome that favoured cognitive therapies: the incidence rate of a first episode of self-harm per person year was 0.584 in the MACT group and 0.71 in the treatment as usual (TAU) group.^20^ The frequency of self-harm events remained relatively unchanged (2.84 per year for MACT versus 2.54 for TAU, after outliers had been excluded). Davidson et al and Palmer et al reported the difference in costs between CBT and TAU, although they were not found to be significant. Palmer et al found CBT to be less costly but also less effective and reported an ICER of £6376 per QALY. When the WTP is below the ICER, CBT has a higher probability of being cost-effective, but when the WTP is above the ICER, TAU has a higher probability of being cost-effective. At the UK threshold of £30 000 per QALY, the probability of CBT being cost-effective was only ~25%, whereas the probability of TAU being cost-effective was ~75%, making TAU the more cost-effective choice. Neither Davidson et al nor Tyrer et al calculated an ICER.

### Stepped care

All three studies on stepped-care interventions used data from RCTs. Sinnaeve et al carried out a CUA, Kvarstein et al employed a CEA and Grenyer et al conducted a CCA.^21–23^ The mean follow-up period was 32 months (s.d. = 18.33 months). The perspective of Kvarstein et al and Sinnaeve et al included healthcare and one other sector's costs (social care and employment respectively), whereas Grenyer et al took the narrowest perspective of all the studies included in the review, only including in-patient stays.

The model of stepped care varied between studies. Grenyer et al considered a brief intervention clinic delivered within 36 h of a crisis presentation, followed by longer-term community-based psychological therapy. A significant reduction in in-patient days was reported. Sinnaeve et al evaluated 3 months of DBT delivered as a residential intervention, followed by 6 months of out-patient DBT, and compared this with 12 months of standard out-patient DBT. Sinnaeve et al reported QALYs with mean (s.d.) utility scores; these were higher for the step-down DBT care group than the comparator group, but this difference was not statistically significant (mean 0.65 (s.d. = 0.33) for stepped-care DBT versus mean 0.62 (s.d. = 0.28) for out-patient DBT). Kvarstein et al evaluated intensive psychotherapy in day hospitals followed by out-patient individual and group therapy. Lower improvements in functioning were reported (measured on the Global Assessment of Functioning scale, GAF) compared with standard out-patient care.

None of the studies reported significant differences in costs. Sinnaeve et al reported an ICER of €278 067 per QALY; at a threshold of €80 000 per QALY there was a 21% likelihood that stepped care was the most cost-effective option. Kvarstein et al reported an ICER for out-patient care compared with stepped care for the subgroup with avoidant personality disorder (but not the subgroup with borderline personality disorder) which was €1092 per additional point gained on the GAF (favouring out-patient care). No uncertainty analysis was reported.

### Nidotherapy

Two studies reported economic evaluations of nidotherapy using data from a single pilot RCT in a population with difficult-to-manage needs, personality disorder diagnoses and other comorbid severe mental illness. Ranger et al evaluated the pilot using a CEA.^24^ Tyrer et al later evaluated nidotherapy in a subgroup of patients with substance misuse problems, employing a CCA using data from the same trial.^25^ The studies used a broad perspective and a follow-up period of 12 months.

Ranger et al^24^ did not observe any significant effects on outcomes, with a trend towards reduced symptoms in the intervention group. Tyrer et al^25^ reported a significant reduction in bed-days in secondary subgroup analyses (54 days compared with 139 for the comparator) but found costs not to be significantly different. Ranger et al still found nidotherapy to be dominant (i.e. it resulted in lower costs and better secondary outcomes than the comparator, although these results were not significant) and even at a threshold of £0 per point of improvement on the Brief Psychiatric Rating Scale it had a 60% likelihood of being cost-effective.

### Schema therapy

Two studies evaluated schema therapy using RCTs, and both conducted a CUA and CEA from a broad perspective.^14,26^ Bamelis et al^14^ compared schema therapy with TAU and van Asselt et al^26^ compared schema therapy with transference-focused psychotherapy. Both studies used the same measure of effectiveness, the proportion of recovered patients, and report this to be higher in the schema therapy group than in the comparator group. For Bamelis et al the proportions were 81.4 *v*. 51.2% respectively (‘recovered’ defined as a score ≤15 on the Structured Clinical Interview for DSM-IV Axis II Disorders version II) and van Asselt et al reported this to be 52% *v*. 29%, respectively (‘recovered’ defined as a score ≤15 on the Borderline Personality Disorder Severity Index). Bamelis et al found that schema therapy produced a greater median gain in number of QALYs than TAU, although this was not significant (2.34 *v*. 2.23; *P* = 0.51). van Asselt et al reported that total QALYs for the schema therapy group was lower than for the transference-focused psychotherapy group, this difference was also not significant (2.15 *v*. 2.27 respectively; 95% CI −0.51 to 0.28). van Asselt et al reported an incremental difference in mean costs: schema therapy was €8969 less costly than transference-focused psychotherapy, although this difference was not significant (95% CI −21 775 to 3546). Bamelis et al also reported a lower mean cost for schema therapy compared with TAU: €23 805 (95% CI 21 014–26 791) *v*. €26 333 (95% CI 22 384–30 605).

Both studies found that schema therapy was dominant with regard to cost per recovered patient and cost per QALY. Bamelis et al showed schema therapy to have an 80% probability of being cost-effective at a WTP threshold of 0, for both cost per recovered patient and cost per QALY. van Asselt et al reported that the likelihood of schema therapy being cost-effective, in terms of recovered patients and QALYs, was over 90% when WTP = 0. Both Bamelis et al and van Asselt et al observed that, as the threshold for recovered patients increased, the probability of schema therapy being cost-effective also increased; however, as the threshold for QALYs increased, the likelihood of schema therapy being cost-effective decreased.

### Interventions defined by setting

Two evaluations conducted by Soeteman et al employed Markov models to evaluate the effect of care being provided in different settings for samples with either cluster B or cluster C ‘personality disorder’ diagnoses,^27^ using data from the SCEPTRE trial.^32,33^ Markov models were used in these CUAs comparing out-patient, day-hospital and in-patient care. One of the studies varied the duration of day-hospital and in-patient care between long and short term.^28^ Both studies adopted a broad perspective, as well as repeating the models taking a narrower, health service provider perspective.

Soeteman et al reported that for a cluster B personality disorder group, care at a day hospital was associated with the greatest QALY gains, and for cluster C personality disorder group, short-term in-patient care produced the greatest estimated number of QALYs. For cluster B patients, out-patient care was the least costly and also dominated other settings (being less costly and more effective). For cluster C patients, short-term day-hospital treatment was the least costly option and dominated all long-term options. Estimated cost-effectiveness acceptability curves (CEAC) for cluster B showed out-patient care to be 84% likely to be cost-effective when the threshold was €0 per QALY. For cluster C patients the estimated CEAC showed short-term in-patient care to be 60% likely to be cost-effective at a high WTP threshold of €80 000 per QALY compared with short-term day-hospital care.

### Other interventions

Four studies were identified which looked at other interventions: joint crisis plans,^29^ psychoeducation with problem-solving,^30^ clarification-oriented psychotherapy (COP)^14^ and mentalisation-based treatment delivered in a day hospital (MBT).^31^ All of them used RCT data and employed a CUA, and two of these studies (Blankers et al^31^ and Bamelis et al^14^) also conducted a CEA. All of these studies took a broad perspective.

Borschmann et al^29^ did not report a significant difference in costs or outcomes. However, their economic analysis found joint crisis plans to be dominant over usual care, with over 80% probability of being cost-effective when the threshold was £0 per QALY. This is not unusual as even in the absence of a significant clinical effect, there can still be a finding of cost-effectiveness. Cost-effectiveness combines point estimates of cost and outcome differences and in some cases, as here, can show high probabilities of interventions being cost-effective. McMurran et al^30^ found that psychoeducation with problem-solving was also dominant, having a 64% chance of being more cost-effective than usual care with a threshold of £20 000–£30 000 per QALY.

Bamelis et al^14^ found COP to be inferior to TAU and schema-focused therapy. Blankers et al^31^ found that MBT was dominated by TAU in the CUA but also that in the CEA the ICER per patient in remission was €29 314. At a threshold of €45 000 the chance of MBT being cost-effective (when considering ‘remission’ as an outcome) was only slight (55%).

### Quality appraisal of studies

Study quality, based on the CHEERS checklist, is detailed in Table 5. There was substantial variation between studies. Of the 18 studies, 10 met over 80% of checklist items. On the basis of this metric, the highest-quality studies were the two that evaluated schema therapy, with both having 95% of items met. The three that evaluated DBT were more varied, with the Priebe et al study^17^ having the highest rating. This was also the case for the three that evaluated CBT, with Palmer et al^19^ scoring highest. The stepped-care evaluations scored the lowest in terms of items met. Overall, studies that scored low tended to do so particularly for reporting of methods.

**Table 5:** Quality appraisal of papers

Study	Title/Abstract (2 items)	Introduction (2 items)	Methods (11 items)	Results (3 items)	Discussion (1 item	Disclosure (2 items)	Total	% of items met
Dialectical behaviour therapy								
Murphy et al^15^	2	2	7	1.5	0.5	0	13	68.4
Pasieczny & Connor^16^	0.5	2	5.5	0.5	1	0	9.5	50.0
Priebe et al^17^	1.5	2	7.5	1.5	1	2	15.5	81.6
Means	1.3	2.0	6.7	1.2	0.8	0.7	12.7	66.7
Cognitive–behavioural therapy								
Davidson et al^18^	1	2	4	2	1	1	11	57.9
Palmer et al^19^	2	1.5	9.5	2	1	1	17	89.5
Tyrer et al^20^	0.5	1.5	6.5	3	0.5	1	13	68.4
Means	1.2	1.7	6.7	2.3	0.8	1.0	13.7	71.9
Stepped care								
Grenyer et al^21^	1.5	2	4.5	1.5	1	2	12.5	65.8
Kvarstein et al^22^	1.5	1.5	5.5	3	1	2	14.5	72.5
Sinnaeve et al^23^	1	1.5	6	1.5	1	2	13	72.2
Means	1.3	1.7	5.3	2.0	1.0	2.0	13.3	70.2
Nidotherapy								
Ranger et al^24^	1.5	0.5	8.5	1	0.5	0.5	12	66.7
Tyrer et al^20^	1	1	8	1	1	2	14	82.4
Means	1.3	0.8	8.3	1.0	0.8	1.3	13.0	74.5
Schema therapy								
Bamelis et al^14^	2	2	10.5	1.5	1	2	19	95.0
van Asselt et al^26^	2	2	10	2	1	2	19	95.0
Means	2.0	2.0	10.3	1.8	1.0	2.0	19	95
Setting								
Soeteman et al^27^	2	2	13.5	2.5	1	0	21	87.5
Soeteman et al^28^	2	2	12.5	2.5	1	2	22	81.7
Means	2.0	2.0	13.0	2.5	1.0	1.0	21.5	89.6
Other								
Blankers et al^31^	2	2	8	1.5	1	2	16.5	82.5
Borschmann et al^29^	1	2	8	2	1	1	15	83.3
McMurran et al^30^	2	2	8	2	0.5	2	16.5	86.8
Means	1.7	2.0	8.0	1.8	0.8	1.7	16.0	84.2

## Discussion

### Summary of main findings

This paper has reviewed economic evaluations of community-based interventions for people with complex emotional needs meeting criteria for personality disorder diagnoses. A diverse range of interventions were identified, with no strong evidence found for the cost-effectiveness of any single intervention or model of care.

The strongest evidence was for dialectical behaviour therapy (DBT) delivered in community settings: all three identified studies indicated that the intervention is likely to be cost-effective compared with treatment as usual (TAU). However, consideration should be given to the limited pool of economic evidence when interpreting this finding. The review also identified evidence to support the use of schema-focused therapy, joint crisis plans, stepped care, nidotherapy, psychoeducation with problem-solving, and manual-assisted cognitive therapy (MACT). The authors with lived experience (E.B. and T.J.) highlighted that the review did not identify any economic evaluations that considered patient-led interventions or workforce development interventions, such as those that focus on therapeutic alliance.

Across all 18 studies the evidence was weakened by small sample sizes or poor quality of evidence. Of the 12 studies that found evidence to favour the intervention evaluated, only 5 reported a statistically significant effect (however, even non-significant effects when combined with cost differences can indicate cost-effectiveness). Of the five studies with significant effects, there were limitations regarding reliability of evidence in at least three (Grenyer et al,^21^ Pasieczny & Connor^16^ and Tyrer et al^25^). Grenyer et al^21^ took a narrow health provider perspective to evaluate their stepped-care intervention, limiting relevance for health policy makers. The drivers for observed differences between groups were unclear and although significant differences in bed-days were observed, there was no difference in admission rates overall (however, reduced bed-days alone may be an important effect). The authors also acknowledge that as there were staff transfers between sites in the cluster RCT design, the reliability of evidence may be limited. Pasieczny & Connor^16^ used a non-randomised trial design, which can lead to biased estimates of effect. Tyrer et al^25^ relied on a subsample of trial participants: unplanned subgroup analysis of trial data can lead to unreliable results by increasing the risk of chance findings.

### Quality of evidence

Although DBT, CBT and stepped care have been the most extensively researched, the numbers of economic evaluations for each of these interventions are relatively few and provide insufficient evidence on which decision makers can confidently base guidelines or allocate resources. This contrasts with other areas, such as depression or schizophrenia, where reasonable agreement about interventions exists. The review found that interventions were sometimes poorly described, limiting reproducibility and usefulness for implementation decisions. Several studies used data from the same trials (BOSCOT^18^^,^^19^ and SCEPTRE^27^^,28^) to report subsequent subgroup analysis or longer-term follow-up. This approach weakens evidence as risk of chance findings is increased and bias may be repeated across more than one study.

### Comparison with other reviews

Although not specifically a review of economic evaluations, Brazier et al^34^ reviewed the literature on treatments for borderline personality disorder and identified six trials from which they then derived cost-effectiveness estimates. In each analysis costs were combined with a parasuicide outcome and in four analyses they also combined costs with QALYs (three of which used QALYs that they mapped from depression scores). A time horizon of 12 months was applied to their models and both a healthcare and broad perspective were taken. Three of the models produced by Brazier et al compared DBT with TAU. From a healthcare perspective and in terms of parasuicide, DBT was dominant in one model, produced a cost per parasuicide event avoided of £40 in another (with lower costs when a broad perspective was taken) and a cost per event avoided of over £40 000 in the third. This was a result of exteremely high incremental costs and a very small QALY gain. This third model also showed a cost per QALY of £273 801. Brazier et al^34^ also modelled the cost-effectiveness of DBT compared to client-centred therapy (CCT), and showed DBT to be dominant both in terms of parasuicide events avoided and QALYs gained. The final two models of Brazier et al^34^ compared TAU with mentalisation-based treatment (MBT) and MACT respectively. MBT was shown to produce a cost per event avoided of £38 and a QALY gained of £7242 and MACT was dominated by TAU in terms of events avoided and had a cost per QALY of £84 032. The authors were clear about the limitations of their analyses. They concluded that there were mixed results for DBT, that MBT had promising results and that MACT could not be considered to be cost-effective. These analyses were interesting and helpful, but now are relatively dated.

A review of both partial (where only costs are compared) and full (costs and outcomes are compared) economic evaluations was conducted by Brettschneider et al.^4^ They included the analyses of Brazier et al^34^ as well as some of the studies included here. Overall, they came to similar conclusions as us, i.e. that the evidence base is limited and that the strongest indications are for DBT.

In a review by Meuldijk et al,^3^ 30 studies were included but some of these were cost comparisons and others were evaluations of hospital-based care. As regards the evaluations (rather than the cost comparisons), they conclude that psychological therapies were likely to be cost-effective although they do recognise that the amount of evidence is greatest for DBT.

Meuldijk et al did include a PhD thesis that was not picked up in our search. This was by Heard and evaluated DBT.^35^ The evaluation consisted of two studies. In the first, DBT was compared with usual care and after 1 year did not differ significantly in terms of total costs or cost-effectiveness. In the second, DBT was compared with ‘stable psychotherapy’. Again, total costs and cost-effectiveness were not significantly different between the groups.

### Limitations

Our search strategy included terms related to community and out-patient care and this may have excluded some relevant studies that did not mention the setting in the title or abstract or where care was delivered in a day service. However, there was good agreement with the other reviews that had been conducted. As has been found in the two previous reviews,^3^^,4^ there was significant heterogeneity in the included studies, which prevented meta-analysis of findings. The results are therefore reported in narrative form, making interpretation of findings more complex. In addition, the comparator was most often ‘usual care’, which is context specific and rarely described in detail, limiting generalisability. The review scope did not intend to include interventions for people with a diagnosis of antisocial personality disorder. However, some of the identified studies considered samples with ‘any personality disorder diagnosis’, another required that patients had other comorbid severe mental illness, and another sample included individuals with complex emotional needs meeting personality disorder diagnostic criteria as well as those not meeting these criteria. Given the general nature of the samples, and because these studies met all other inclusion criteria, they were included in the review. However, this presents a key limitation for identifying and interpreting findings specific to the population of interest and highlights that diagnostic categorisation, although essential for literature searching, is a limitation of the review scope. Finally, to simplify reporting, we assigned a quality assessment score to each paper, which may risk oversimplifying quality assessment, where it is important to understand the areas of strength and weakness. To address this limitation, we have provided detail on areas of quality assessed (Table 5).

### Recommendations for future research

The majority of included studies relied on data from small RCTs. RCTs are often seen as the gold standard of evidence on the efficacy of treatment. Evidence on effectiveness can also be generated through trials but these usually need to be sufficiently large to capture real-world effects. Inclusion of these effects can be informative for decision-making as results are often more generalisable. Economic modelling can also support decision-making and allow for uncertainty in results to be readily examined. Given the scarcity of economic evidence in this area, observational studies alongside the delivery of community services would be valuable. We did not identify such studies in our review but some may exist in the form of reports to specific organisations.

The review found that, although a range of economic approaches were used, studies using CUAs applied the most rigorous and transferable methods. The follow-up periods were longer, the perspectives were broader and resource use was more often collected through a combination of patient report and patient records, improving the accuracy of healthcare utilisation estimates. The main benefit of CUAs is that they enable comparison across disease areas by measuring effect in QALYs. All CUAs in this review used EuroQuol's EQ-5D to derive QALYs (in line with NICE guidelines). The EQ-5D has been criticised for not picking up all important aspects of health, and so for all health conditions consideration must be given to its reliability (does it produce consistent results?), validity (does it measure what it intends to measure?) and sensitivity (does it identify genuine changes in health states?). The EQ-5D has been shown to be moderately responsive to change in symptoms in individuals with personality disorder diagnoses.^36^ There is a lack of evidence on its validity in capturing all important aspects of health in this population.^34^ Nonetheless, owing to the measure's simplicity and because it can be used to generate QALYs, it has been recommended as appropriate for use in this population.^36^

A limitation of CUAs is that QALYs singularly focus on health benefits, which prevents comparisons across sectors (e.g. comparing whether an education intervention may be more cost-effective than a health intervention) and may underestimate benefits where interventions have wider outcomes such as employment. The majority of studies included in this review took a broad perspective in measuring costs and effects. Five studies also used CCAs, presenting costs alongside a number of outcomes. Although this can make interpretation challenging, CCAs may be justified, given the multifaceted nature of the conditions being studied and the potential breadth of effects. A broad perspective and presenting multiple outcomes alongside QALYs may therefore be appropriate where interventions aim to improve outcomes beyond health gain and where cost implications may fall outside of the health and care budget.

Future research should aim to co-produce studies with people with lived experience of diagnoses of personality disorder or complex emotional needs to help ensure that important outcomes, as well as costs, are captured from a broad perspective. Choice of outcome measures should also be informed by previous studies and local guidelines. Consistency in measurement and reporting will support the development of a stronger evidence base for community-based interventions as information can be pooled across multiple studies. Researchers may wish to consult the ICHOM Standard Set for Personality Disorders when selecting measures.^37^

Future studies should only evaluate therapies that are well developed and hold face validity with people who are using and delivering services, and they should be based on sound theoretical foundations. High-quality research is also needed on models of care, including the intensity and duration of interventions, with clear description of how services are configured to enable reproducibility. Researchers may wish to refer to a forthcoming paper by Finamore et al ‘Systematic scoping of community-based service models for people with personality disorder’ (personal communication, 2021; further details are available from P.M. on request) to support a standardised description and understanding of models of care. The logic models articulated in that paper may also support the identification of relevant outcome measures. Finally, the review identified a gap in the types of intervention evaluated, with no patient-led interventions or workforce interventions identified. These two areas should be considered key areas for future economic research.

### Lived experience commentary by Eva Broeckelmann

‘Having spent years struggling to access suitable treatment for CEN [complex emotional needs], I am pleased to see the lack of robust economic evidence to support a *single* intervention or model of community-based care.

Especially considering the inherently heterogeneous nature of any given sample with a ‘PD’ [personality disorder] diagnosis – where e.g. two people with ‘BPD’ [bipolar personality disorder] may have no more than one highly subjective trait in common – there simply is no ‘one-size-fits-all’ approach. Therefore, any study results must be treated with utmost caution before making generic policy decisions that indiscriminately apply to everyone with this label.

Despite being recommended by NICE, I do not consider the EQ-5D to be an appropriate outcome measure for this population. CEN symptoms can fluctuate so frequently that any snapshot in time on such a rudimentary measure is virtually meaningless for assessing long-term recovery, and it will be crucial to develop more nuanced alternatives for future studies.

Ultimately, the policy aim to prioritise specific interventions based on cost-effectiveness neglects the fact that strong therapeutic relationships with trusted clinicians are considerably more important for treatment success than the particular modality used. Thus, such comparisons are of limited value, whereas it could eventually be much more cost-effective to focus research and resources on improved training for clinicians instead.’

### Lived experience commentary by Tamar Jeynes

‘It is interesting that in life much interacts with and mirrors itself. This systematic review endeavoured to be robust: 18 economic evaluations in 19.5 years fit the inclusion criteria. Nine different interventions, each with their own access criteria for service users.

Collecting robust economic data involves resource. This luxury is afforded to better funded interventions, which then make a case for future funding. Many service users do not meet criteria for these interventions.

It is interesting to identify what is missing.

There are no economic evaluations of survivor-led or co-produced interventions, such as co-facilitation of therapies, survivor-led networks or crisis houses. These are more likely to not have inclusion criteria, reaching people that other interventions cannot. They often do not have the resource to conduct economic evaluations. Many have ceased to exist. Excluded service users only have access to costly emergency services during crisis. Being excluded depletes the internal resources needed to value their worth. Many cease to exist.

It is interesting that in life much interacts with and mirrors itself. When an intervention cannot demonstrate its worth, it cannot access funding, which includes resource to measure its worth. When the only interventions that can demonstrate their worth are ones with inclusion criteria, excluded service users remain without a service.

The cycle continues.’

## Data Availability

Data availability is not applicable to this article as no new data were created or analysed in this study.

## Supplementary material

For supplementary material accompanying this paper visit http://dx.doi.org/10.1192/bjo.2021.1043. click here to view supplementary material

## Appendix

### Inclusion criteria

- Studies that include an economic evaluation where both costs and outcomes are reported and where a formal or informal link between costs and outcomes is made. This will include trials, observational studies and models
- The population of interest are adults with complex emotional needs who are in contact with community mental health services. This will include evaluations of services for those with a diagnosis of personality disorder (excluding antisocial) or services focused specifically on trauma-related care
- Evaluations of community-based services/treatments/interventions
- Evaluations with usual care (which may include ‘do nothing’ or waiting lists) or another active intervention as a comparator

### Exclusion criteria

- Studies that are only reported as conference abstracts, letters, protocols, reviews or editorials
- Evaluations of interventions only provided in in-patient or forensic settings
- Evaluations of pharmacological interventions
- Studies that do not report costs
- Studies that do not report outcomes
- Studies with no comparator
- Studies not reported in English
