# Supplementary material for: Community interventions for people with complex emotional needs that meet the criteria for personality disorder diagnoses: systematic review of economic evaluations and expert commentary
Source: BJPsych Open. 2021 Nov 15;7(6):e207. doi: 10.1192/bjo.2021.1043 (PMC8612014; doi:10.1192/bjo.2021.1043)
Supplement: Supplementary file 1 [file bjosup.zip › S2056472421010437sup001.docx]

**Appendix**

**Search Terms**

| **Search** | Embase | MEDLINE | Global Health* | PsychINFO* |
| --- | --- | --- | --- | --- |
| (borderline state or borderline person$).sh. | 13128 | 6551 | 0 | 6280 |
| borderline$.mp. | 65471 | 42211 | 5495 | 21372 |
| exp personality disorders/ | 57394 | 40349 | 0 | 0 |
| (borderline$ adj3 (disorder$ or person$ or PD$1 or state$)).tw. or (borderline$ and personalit$).mp. | 12880 | 10377 | 274 | 15494 |
| (borderline$ and cluster b).mp. | 339 | 211 | 9 | 316 |
| (emotion$ adj2 (instabil$ or unstable) adj3 (character$ or difficult$ or disorder$ or dysfunction$ or PD or person$1 or personalit$ or state$)).tw. | 266 | 168 | 11 | 217 |
| (multiple personality disorder$ or personality disorder$).sh. | 27026 | 19759 | 0 | 11799 |
| (personalit$ adj (disorder$ or dysfunction$)).tw. | 25798 | 19156 | 738 | 32911 |
| (dsm and (axis and II)).mp. | 2152 | 1445 | 63 | 7049 |
| 1 or 2 or 3 or 4 or 5 or 6 or 7 or 8 or 9 | 113361 | 80310 | 6055 | 47812 |
| exp Economics/ | 247804 | 583602 | 24814 | 69855 |
| exp "Costs and Cost Analysis"/ | 338196 | 227858 | 0 | 0 |
| exp Economics, Dental/ | 813840 | 4056 | 0 | 0 |
| exp Economics, Hospital/ | 813840 | 23830 | 0 | 0 |
| exp Economics, Medical/ | 813840 | 14121 | 0 | 0 |
| exp Economics, Nursing/ | 813840 | 3990 | 0 | 0 |
| exp Economics, Pharmaceutical/ | 196408 | 2886 | 0 | 0 |
| (economic$ or cost or costs or costly or costing or price or prices or pricing or pharmacoeconomic$).ti,ab. | 970752 | 740897 | 172960 | 205378 |
| (expenditure$ not energy).ti,ab. | 37952 | 28106 | 8567 | 7733 |
| value for money.ti,ab. | 2261 | 1597 | 388 | 478 |
| budget$.ti,ab. | 36340 | 27809 | 5561 | 8361 |
| 11 or 12 or 13 or 14 or 15 or 16 or 17 or 18 or 19 or 20 or 21 | 1608980 | 1161813 | 188841 | 249177 |
| exp Outpatients/ | 113649 | 14913 | 0 | 0 |
| outpatient$.ti,ab,kw. | 256292 | 158994 | 24631 | 51753 |
| exp Community Mental Health Services/ | 53063 | 18229 | 0 | 0 |
| (community adj2 (mental or health$ or care) adj3 (service$ or team$)).ti,ab,kw. | 7412 | 5585 | 1341 | 3719 |
| exp Community Health Services/ | 112037 | 292611 | 3726 | 0 |
| (community adj5 (support or mental health or model or service or treatment or care or day or week$ or nurse)).ti,ab,kw. | 83155 | 63502 | 14427 | 43784 |
| (network or outreach or ((specialist or day or whole) adj3 service)).ti,ab,kw. | 435878 | 342289 | 25009 | 91655 |
| 23 or 24 or 25 or 26 or 27 or 28 or 29 | 909533 | 821044 | 74912 | 181965 |
| 10 and 22 and 30 | 657 | 264 | 14 | 173 |

* denotes term “kw” not searched.

NHS EED

| (borderline state) OR (borderline person*) IN NHSEED | 10 |
| --- | --- |
| (borderline*) IN NHSEED | 60 |
| MeSH DESCRIPTOR Personality Disorders EXPLODE ALL TREES IN NHSEED | 18 |
| (borderline* adj3 (disorder* or person* or PD* or state*)) IN NHSEED | 10 |
| (borderline*) AND (cluster b) IN NHSEED | 0 |
| (emotion* adj2 (instabil* or unstable) adj3 (character* or difficult* or disorder* or dysfunction* or PD or person* or personalit* or state*)) IN NHSEED | 0 |
| (multiple personality disorder* or personality disorder*) IN NHSEED | 27 |
| (personalit* adj (disorder* or dysfunction*)) IN NHSEED | 28 |
| (DSM) AND ((axis and II)) IN NHSEED | 3 |
| #1 OR #2 OR #3 OR #4 OR #5 OR #6 OR #7 OR #8 OR #9 | 80 |
