# Supplementary material for: Community interventions for people with complex emotional needs that meet the criteria for personality disorder diagnoses: systematic review of economic evaluations and expert commentary
Source: BJPsych Open. 2021 Nov 15;7(6):e207. doi: 10.1192/bjo.2021.1043 (PMC8612014; doi:10.1192/bjo.2021.1043)
Supplement: Supplementary file 1 [file bjosup.zip › S2056472421010437sup002.docx]

**Supplementary information: Interventions included in studies**

| **Intervention** | **Description** | **Number of studies identified** | **Study** |
| --- | --- | --- | --- |
| **Dialectical behavioural therapy** | A form of psychotherapy adapted from cognitive behavioural therapy. Includes individual therapy, group skills training, phone coaching and consultation meetings for clinicians. | 3 | Murphy et al (2019)  Pasieczny & Connor (2011)  Priebe et al (2012) |
| **Cognitive behavioural therapy** | Structured, time limited, psycho-social intervention focussing on practical goals to address problems with social functioning. | 2 | Palmer et al (2006)  Davidson et al (2010) |
| **Manual-assisted cognitive therapy** | Brief focused therapy for people with repeated incidents of self-harm. Service users receive a 70-page booklet and are offered up to seven sessions with a therapist which focus on methods to reduce distress and resolve problems. | 1 | Tyrer et al (2004) |
| **Clarification orientated psychotherapy** | Open ended client-centred psychotherapy addressing dysfunctional interaction behaviours delivered via individual outpatient sessions. | 1 | Barnelis et al (2015) |
| **Nidotherapy** | Systematic assessment and modification of a service user’s physical, social and personal environment through agreed set of targets. | 2 | Ranger et al (2009)  Tyrer et al (2011) |
| **Schema-focused therapy** | Integrative cognitive therapy combining experiential, behavioural and interpersonal therapy. | 2 | Barnelis et al (2015)  Van Asselt et al (2008) |
| **Transference-focused psychterapy** | Psychodynamically based psychotherapy. | 1 | Van Asselt et al (2008) |
| **Psycho-education with problem solving** | Up to four psycho-education sessions discussing diagnosis of ‘personality disorder’ to improve knowledge. Build rapport  and motivate participants. Problem-solving therapy delivered via 12 group sessions aiming to help people learn a strategy for solving interpersonal problems. | 1 | McMurran et al (2016) |
| **Mentalisation based therapy in a day hospital setting** | Intensive day hospitalisation for minimum of 18 months including daily group psychotherapy, weekly individual psychotherapy and individual crisis planning alongside social and community meetings and other activities such as art therapy. Followed by 18 months group therapy. | 1 | Blankers et al (2019) |
| **Interventions defined by setting** | Out-patient, day hospital and in-patient psychotherapy or psychosocial services. Duration of treatment also explored. | 2 | Soeteman et al (2010)  Soeteman et al (2011) |
| **Stepped-care approach** | Intensive day, residential or outpatient intervention followed by step down to longer term outpatient/community follow-up psychotherapy. | 3 | Sinnaeve et al (2018)  Kvarstein et al (2013)  Grenyer et al (2018) |
| **Joint crisis plans** | Written documentation of treatment preferences for management of future crises. Developed between service user and clinician with facilitation from independent mental health professional. | 1 | Borschmann et al (2013) |

Bamelis LLM, Arntz A, Wetzelaer P, Verdoorn R, Evers SMAA. Economic evaluation of schema therapy and clarification-oriented psychotherapy for personality disorders: A multicenter, randomized controlled trial. J Clin Psychiatry. 2015 Nov 1;76(11):e1432–40.

Blankers M, Koppers D, Laurenssen EMP, Peen J, Smits ML, Luyten P, et al. Mentalization-Based Treatment Versus Specialist Treatment as Usual for Borderline Personality Disorder: Economic Evaluation Alongside a Randomized Controlled Trial With 36-Month Follow-Up. J Pers Disord. 2019 Nov 4;1–20.

Borschmann R, Barrett B, Hellier JM, Byford S, Henderson C, Rose D, et al. Joint crisis plans for people with borderline personality disorder: Feasibility and outcomes in a randomised controlled trial. Br J Psychiatry. 2013 May;202(5):357–64.

Davidson KM, Tyrer P, Norrie J, Palmer SJ, Tyrer H. Cognitive therapy v. usual treatment for borderline personality disorder: Prospective 6-year follow-up. Br J Psychiatry. 2010 Dec;197(6):456–62.

Grenyer BFS, Lewis KL, Fanaian M, Kotze B. Treatment of personality disorder using a whole of service stepped care approach: A cluster randomized controlled trial. PLoS One. 2018 Nov 1;13(11).

Kvarstein EH, Arnevik E, Halsteinli V, Rø FG, Karterud S, Wilberg T. Health service costs and clinical gains of psychotherapy for personality disorders: A randomized controlled trial of day-hospital-based step-down treatment versus outpatient treatment at a specialist practice. BMC Psychiatry. 2013 Nov 22;13.

McMurran M, Crawford MJ, Reilly J, Delport J, McCrone P, Whitham D, et al. Psychoeducation with problem-solving (PEPS) therapy for adults with personality disorder: A pragmatic randomised controlled trial to determine the clinical effectiveness and cost-effectiveness of a manualised intervention to improve social functioning. Health Technol Assess (Rockv). 2016 Jul 1;20(52):1–249.

Murphy A, Bourke J, Flynn D, Kells M, Joyce M. A cost-effectiveness analysis of dialectical behaviour therapy for treating individuals with borderline personality disorder in the community. Ir J Med Sci. 2019

Palmer S, Davidson K, Tyrer P, Gumley A, Tata P, Norrie J, et al. The cost-effectiveness of cognitive behavior therapy for borderline personality disorder: Results from the BOSCOT trial. J Pers Disord. 2006 Oct;20(5):466–81.

Pasieczny N, Connor J. The effectiveness of dialectical behaviour therapy in routine public mental health settings: An Australian controlled trial. Behav Res Ther. 2011 Jan;49(1):4–10.

Priebe S, Bhatti N, Barnicot K, Bremner S, Gaglia A, Katsakou C, et al. Effectiveness and Cost-Effectiveness of Dialectical Behaviour Therapy for Self-Harming Patients with Personality Disorder: A Pragmatic Randomised Controlled Trial. 2012;

Ranger M, Tyrer P, Miloseska K, Fourie H, Khaleel I, North B, et al. Cost-effectiveness of nidotherapy for comorbid personality disorder and severe mental illness: Randomized controlled trial. Epidemiol Psichiatr Soc. 2009;18(2):128–36.

Sinnaeve R, van den Bosch LMC, Hakkaart-van Roijen L, Vansteelandt K. Effectiveness of step-down versus outpatient dialectical behaviour therapy for patients with severe levels of borderline personality disorder: A pragmatic randomized controlled trial. Borderline Personal Disord Emot Dysregulation. 2018 Jul 10;5(1).

Soeteman DI, Verheul R, Meerman AMMA, Ziegler U, Rossum B V., Delimon J, et al. Cost-effectiveness of psychotherapy for cluster C personality disorders: A decision-analytic model in The Netherlands. J Clin Psychiatry. 2011 Jan;72(1):51–9.

Soeteman DI, Verheul R, Delimon J, Meerman AMMA, Van Den Eijnden E, Rossum B V., et al. Cost-effectiveness of psychotherapy for cluster B personality disorders. Br J Psychiatry. 2010 May;196(5):396–403.

Tyrer P, Tom B, Byford S, Schmidt U, Jones V, Davidson K, et al. Differential effects of manual assisted cognitive behavior therapy in the treatment of recurrent deliberate self-harm and personality disturbance: The popmact study. Vol. 18, Journal of Personality Disorders. 2004. p. 102–16.

Tyrer P, Milošeska K, Whittington C, Ranger M, Khaleel I, Crawford M, et al. Nidotherapy in the treatment of substance misuse, psychosis and personality disorder: Secondary analysis of a controlled trial. Psychiatrist. 2011 Jan;35(1):9–14.

Van Asselt ADI, Dirksen CD, Arntz A, Giesen-Bloo JH, Van Dyck R, Spinhoven P, et al. Out-patient psychotherapy for borderline personality disorder: Cost-effectiveness of schema-focused therapy v. transference-focused psychotherapy. Br J Psychiatry. 2008 Jun;192(6):450–7.
